# Synergistic Anti-inflammatory and Neuroprotective Effects of *Cinnamomum cassia* and Z*ingiber officinale* Alleviate Diabetes-Induced Hippocampal Changes in Male Albino Rats: Structural and Molecular Evidence

**DOI:** 10.3389/fcell.2021.727049

**Published:** 2021-09-08

**Authors:** Hailah M. ALmohaimeed, Zuhair M. Mohammedsaleh, Ashwaq H. Batawi, Maha Jameal Balgoon, Osama Ibrahim Ramadan, Hasan Ahmed Baz, Soad Al Jaouni, Nasra Naeim Ayuob

**Affiliations:** ^1^Department of Basic Science, Medical College, Princess Nourah Bint Abdulrahman University (PNU), Riyadh, Saudi Arabia; ^2^Department of Medical Laboratory Technology, Faculty of Applied Medical Sciences, University of Tabuk, Tabuk, Saudi Arabia; ^3^Department of Biological Science, Faculty of Science, King Abdulaziz University, Jeddah, Saudi Arabia; ^4^Department of Biochemistry, Faculty of Science, King Abdulaziz University, Jeddah, Saudi Arabia; ^5^Histology Department, Damietta Faculty of Medicine, Al Azhar University, Cairo, Egypt; ^6^Clinical Toxicology, Faculty of Medicine, Umm Al Qura University/King Abdullah Medical City, Mecca, Saudi Arabia; ^7^Department of Hematology/Pediatric Oncology, Yousef Abdul Latif Jameel Scientific Chair of Prophetic Medicine Application, Faculty of Medicine, King Abdulaziz University, Jeddah, Saudi Arabia; ^8^Medical Histology and Cell Biology Department, Faculty of Medicine, Damietta University, Damietta, Egypt

**Keywords:** diabetes mellitus, depression, ginger, cinnamon, corticosterone, BDNF, apoptosis

## Abstract

**Background:** Depression has been reported as a common comorbidity in diabetes mellitus although the underlying mechanism responsible for this is not well known. Although both ginger and cinnamon has anti-diabetic, antioxidant, and neuroprotective properties, their efficacy in inhibiting neuroinflammation, when simultaneously administrated, has not been investigated yet.

**Objectives:** The study was designed to assess the synergistic effect of *Cinnamomum cassia* and Z*ingiber officinale* on regulating blood glucose, improve hippocampal structural changes and depressive-like alternations in diabetic rats, and try to identify the mechanism behind this effect.

**Materials and Methods:** Thirty male Sprague–Dawley rats were divided into five equal groups (*n* = 6): the normal control, untreated streptozotocin (STZ)-diabetic, cinnamon-treated diabetic [100 mg/kg of body weight (BW)/day for 6 weeks], ginger-treated diabetic (0.5 g/kg BW/day for 6 weeks), and ginger plus cinnamon-treated diabetic groups. Forced swim test and elevated plus maze behavioral tests were performed at the end of the experiment. HOMA-IR, HOMA β-cells, blood glucose, insulin, corticosterone, pro-inflammatory cytokines tumor necrosis factor-α (TNF-α) and IL-6), and total anti-oxidant capacity (TAC) were assessed in the serum. BDNF mRNA level was assessed in hippocampus using qRT-PCR. Hippocampal histopathological changes were also assessed, and immunoexpression of glial fibrillary acidic protein (GFAP), caspase-3, and Ki-67 was measured.

**Results:** Diabetes-induced depressive-like changes in the STZ group were biochemically confirmed by assessing serum corticosterone level, as well as behaviorally using FST and EPM tests. Diabetes also induced degenerative changes in the hippocampus. Treatment of diabetic rats with ginger, cinnamon, or the combination of these alleviated the degenerative structural changes and significantly up-regulated serum insulin, TAC, hippocampal BDNF mRNA, and hippocampal immunoexpression of ki67, while they significantly reduced serum blood glucose, IL-6, TNF-α, IL1β, as well as hippocampal immunoexpression of GFAP and Caspase-3 compared to the untreated diabetic group. Improvement induced by the combination of ginger and cinnamon was superior to the single administration of either of these.

**Conclusion:***Cinnamomum cassia* and Z*ingiber officinale* have synergistic anti-diabetic, antioxidant, anti-inflammatory, antidepressant-like, and neuroprotective effects. The use of a combination of these plants could be beneficial as alternative or complementary supplements in managing DM and decreasing its neuronal and psychiatric complications.

## Introduction

There is an increasing global prevalence of diabetes mellitus (Russell et al., 1999) as over 400 million people around the world currently suffer from this metabolic disease (Lee et al., 2021). The burden of DM is becoming increasingly heavy worldwide. Diabetes mellitus can lead to many complications affecting the cardiovascular, nervous, renal, and other body systems which puts a significant burden on healthcare systems globally (Alwafi et al., 2020).

Hippocampus, the center for learning and memory, is sensitive to high blood glucose and undergoes structural, neurophysiological, and molecular changes during diabetes. Diabetes-related hyperglycemia was reported to increase oxidative stress and neuronal death and reduce neurogenesis (Heidarianpour et al., 2021). Structural and functional changes were also described in the brain, especially in the hippocampus, during uncontrolled diabetes (Hasanein et al., 2020). Poor glycemic control and functional impairment due to increasing diabetes complications may cause or worsen depression and anxiety in diabetic patients (Khan et al., 2019).

It was reported that blood-brain barrier compromise in DM type 1 and 2 *in vivo* models is associated with memory deficits, gene alterations in brain endothelium, and neuroinflammation (Rom et al., 2019). Therefore, prevention, early detection, and treatment of diabetes-related depression are essential for the optimal management of diabetes and for improving patients’ overall quality of life. Regarding the structural changes associated with diabetes, it was reported that apoptosis plays a crucial role in diabetes-induced neuronal loss in hippocampus. They found that DM induces hippocampal neuronal apoptosis through oxidative stress, disturbing the expression of apoptosis regulator genes and the induction of deficits in mitochondrial function (Sadeghi et al., 2016).

Hypoglycemia and insulin resistance are among the side effects of the conventional, widely used anti-diabetic treatment including oral hypoglycemic drugs and insulin injections (Jayaraman et al., 2018). Therefore, limitations of the currently available anti-diabetic drugs and diabetes-associated morbidity and mortality as well as increased DM prevalence highlight the importance of searching for effective, safe, preventive, and treatment strategies of DM (Abdi, 2021). The Homeostatic model assessment (HOMA) model has proved be a robust clinical and epidemiological tool in descriptions of the pathophysiology of diabetes. HOMA analysis allows the assessment of inherent β-cell function and insulin sensitivity and can characterize the pathophysiology in those with abnormal glucose tolerance (Wallace et al., 2004).

Herbal medicines are more popular due to its safety and efficacy during treatment. To date, several herbal medicines have been targeted in depth pathophysiology of diabetes mellitus through anti-inflammatory, antioxidant, antihyperlipidemic, and antihyperglycemic mechanisms (Wasana et al., 2021). Ginger (*Zingiber officinale* Roscoe) belongs to the Zingiberaceae family and is widely used in Southeast Asian countries especially in the Indian medicinal system for its anti-inflammatory, anti-diabetic, and antioxidant effects. *Cinnamon* (*Cinnamomum cassia* L.) is considered an alternative natural medicine that has been traditionally used for treating urinary tract infections, relieving abdominal discomfort, and improving digestion, adding to its analgesic, anti-diabetic, antioxidant, and neuroprotective effects (Sharifi-Rad et al., 2021). The neurotropic property of cinnamon, specifically on the brain-derived neurotropic factor (BDNF), was previously reported and proposed to be of benefit for various neurodegenerative disorders (Jana et al., 2013). Ginger also showed a neuroprotective activity, evidenced by upregulation of BDNF, with anti-oxidative and anti-inflammatory properties in traumatic brain injury (Sahin et al., 2019).

Although both ginger and cinnamon, separately, have many beneficial effects in controlling DM, the effect of their combined administration was not fully investigated as a potential candidate in ameliorating the pathological sequels of neurological complications of diabetes. Therefore, this study aimed to assess the synergistic effect of *Cinnamomum cassia* and Z*ingiber officinale* on glucose hemostasis and diabetes-induced structural changes in rat hippocampus and to explore the mechanism behind this effect. The study also aimed at exploring the antidepressant-like effect of these two plants when administrated together in diabetic rats. It is hypothesized, in this study, that oral administration of *Cinnamomum cassi*a and *Zingiber officinale* might have higher anti-hyperglycemic and antioxidant effects in diabetic rats, exert an antidepressant-like effect, and preserve the hippocampus better than the single administration of either plant extract. This synergistic effect of *Cinnamomum cassia* and Z*ingiber officinale* might be mediated through downregulation of pro-inflammatory cytokines and upregulation of the brain-derived neurotropic factor (BDNF).

## Materials and Methods

### Extraction of Ginger and Dosage

Fresh roots of ginger (*Zingiber officinale* Roscoe) (voucher specimen: AQJ_84) and cinnamon (*Cinnamomum cassia*, L) (voucher specimen: AQJ_15) were purchased from the local market at Jeddah, Saudi Arabia. They were identified in the King Abdulaziz University herbarium using specimens of herbarium, Flora of Kingdom of Saudi Arabia (Chaudhary, 2001). Voucher specimens were deposited in the herbarium, and the identification of the plants was verified by a botanist from the Faculty of Science, KAU.

The watery extract of ginger was prepared as previously described by Al-Amin et al. (2006) and stored in a refrigerator at 4°C. It was dissolved in normal saline and orally administrated through a gastric gavage at a dose of 500 mg/kg of body weight per day for 6 weeks. This dose was selected as it was reported to induce a significant impact when orally administrated (Al-Amin et al., 2006).

### Extraction of Cinnamon and Dosage

The aqueous extract of cinnamon was prepared according to the method previously described (Longe et al., 2015). It was dissolved in normal saline and orally administrated through the gastric gavage at the dose of 100 mg/kg of body weight per day for 6 weeks, as this dose was reported to induce a significant hypoglycemic effect of cinnamon (Longe et al., 2015).

### Experimental Design and Drug Administration

This study was approved by the biomedical research ethics committee, at the Faculty of Medicine, King Abdulaziz University, Jeddah, Saudi Arabia. Thirty male Sprague–Dawley rats with average weight ranging from 100 to 150 g and age of 5 weeks ± 3 days were obtained from King Fahed Medical Research Center (KFMRC), King Abdulaziz University, Jeddah, Saudi Arabia. Rats were kept for 1 week to acclimatize under the standard laboratory conditions with free access to standard animal chow and water. Streptozotocin (STZ) (Sigma Aldrich Chemical Company, Co., St. Louis, MO, United States) was intraperitoneally injected in 12-h-fasted rats followed by a 5% sucrose solution for the induction of diabetes at a dose of 60 mg/kg BW dissolved in 0.01 M sodium citrate buffer, pH 4.5 (Salemi et al., 2016).

The rats were divided into five groups (*n* = 6 each): the control group (CON) that was given normal saline using a gastric tube and four groups that received freshly prepared STZ for induction of DM. The latter was confirmed by measuring the blood glucose level (BGL) after 1 week of STZ injection, and rats with BGL of 250 mg/dl or more were considered diabetic (Nie et al., 2002). The four diabetic groups (*n* = 6 each) included: the untreated (STZ), cinnamon-treated (STZ + Cinn), ginger-treated (STZ + Gin), and ginger plus cinnamon-treated (STZ + Cinn + Gin) groups. After completing 6 weeks of treatment, blood was obtained from the retro-orbital vein of all rats after being fasted for 12 h and centrifuged for 15 min at 3,000 rpm, and the serum was kept at −80°C for biochemical assessment.

### Evaluation of Changes in Rat Behavior

The depressive-like effect of DM in rats was assessed using a forced swim test (FST) and elevated plus maze (EPM) that were performed after completing 6 weeks of treatment according to the previously described methods (Yankelevitch-Yahav et al., 2015; Ali et al., 2017). After a preswim session that was performed 24 h before the test session, each rat was left to swim, during FST, in a glassy cylindrical container filled with water at 25 ± 2°C and was observed for 6 min by a technician. The total immobility time, in which the rat was immobile during the 6 min, was recorded in seconds. Immobility was defined as “the cessation of limb movement, except for the minor movement necessary to keep the rat floating.”

The EPM is considered a validated widely used behavioral assay in rodents to assess the anti-anxiety effects of pharmacological agents and to study the mechanisms underlying anxiety-related behavior (Walf and Frye, 2007). The number of closed arm entries in 6 min, during EPM, and the time spent by each rat in the open arm was assessed in seconds.

### Biochemical Assessment

Blood glucose level was assessed using enzymatic kits (Human Gesellschaft für and Diagnostica mbH, Germany), while serum insulin was assessed using ELISA kits (Cat. no. ezrmi-13kelisa, Billerica, MA, United States) according to the manufactures instruction.

The HOMA model for assessing insulin resistance (HOMA-IR) and for assessing β-cells (HOMA β-cells) was calculated as previously described based on these formulas: HOMA-IR = fasting serum glucose (mg/dl) × fasting serum insulin (μU/ml)/405. HOMA-β cell function = 20 × fasting serum insulin (μU/ml)/fasting serum glucose (mg/dl) −3.5 (Matthews et al., 1985).

Corticosterone level (ALPCO Diagnostics, Orangeburg, NY, United States) was measured in the serum using ELISA kits according to the manufacturers’ instructions. The total antioxidant capacity (TAC) was estimated using the Bio-diagnostic kit method (Koracevic et al., 2001).

### Assessment of Gene Expression of Pro-inflammatory Cytokines

At the end of the experiment, rats were euthanized by cervical dislocation after being anesthetized with 4% isoflurane (SEDICO Pharmaceuticals Company, Cairo, Egypt) in 100% oxygen. The brain was quickly dissected out, fixed in 10% neutral buffered formalin for 24 h, and processed for obtaining paraffin blocks.

Parts of paraffin-processed hippocampal tissue were obtained from the left hemisphere of the brain and underwent RNA as was previously described (Pikor et al., 2011). Extraction of total RNA using Trizol was performed based on the supplier instruction (Invitrogen Life Technologies, Carlsbad, CA, United States). Nano Drop 2,000 Spectrophotometer (Thermo Fisher Scientific, Carlsbad, CA, United States) was used to measure the concentration of RNA. Reverse transcription was done using oligo-dT primers (Bioneer Inc., Daejeon, South Korea) in a 20-ll reaction including 5 ll RNA. Obtained cDNAs were amplified using PCR Master Mix (Bioneer Inc.) with primers (Metabion International AG, Semmelweisstr, Germany) (Pikor et al., 2011).

The results analysis was performed using the Light Cycler 480 software (version 1.5, Roche Applied Science, Mannheim, Germany). The relative levels of mRNA were analyzed using the ΔΔCt method. For GAPDH (forward) CAACTCCCTCAAGATTGTCAGCAA-3′, (backward) 5′-GGCATGGACTGTGGTCATGA-3′; for tumor necrosis factor-α (TNF-α) 5′-CCCTGGTACTAACTCCCAGAAA-3′ (forward) and 5′-TGTATGAGAGGGACGGAACC-3′ (backward); for IL-1β 50-AGTAAGTTCCTCTCTG CAAGAGACT-30 (forward) and 50-CACTAGGTTTGCCGAGTAGAT CTC-30 (backward); for IL-6 5′-CTGCAAGAGACTTCCATCCAG-3′ (forward), 5′-AGTGGTATAGACAGGTCTGTTGG-3′ (backward); for BDNF (forward) 5′-TATTTCATACTTCGGTTGC-3′, (reverse) 5′-TGTCAGCCAGTGATGTCG-3′).

### Assessment of Histological Changes

After being processed into paraffin blocks, the right hemisphere of the brain was sectioned and 4-μm-thick sections were obtained and stained with hematoxylin and eosin (H&E). Immunohistochemical staining with the streptavidin–biotin–peroxidase technique was also performed using anti-glial fibrillary acidic protein (GFAP) (Dako Cytomation, Carpinteria, CA, United States at a dilution 1:1,000), a specific maker of astrocytes; anti-Caspase-3 (Santa Cruz Biotechnology, Santa Cruz, CA, United States at a dilution of 1:1,000), a marker of apoptosis; and anti-Ki-67 antibody (Abcam, Cambridge, United Kingdom, at a dilution of 1:100), an indirect marker of neural cell proliferation. The color was developed in these sections using diaminobenzidine tetrahydrochloride supplemented with 0.04% hydrogen peroxidase and counterstained with Mayer’s hematoxylin.

A light microscope BX-51 (Olympus) connected to a digital camera was used for histopathological examination of the slides by a histologist blinded to the study groups. The Pro Plus image analysis software was utilized for semi-quantitative assessment of the immunoexpression of the studied antibodies. Caspase-3-positive cells were counted per 1.0 mm^2^ of hippocampal CA3 subregion and dentate nucleus while the area percentage of GFAP immunoexpression was assessed as previously described (Zhou et al., 2017). A minimum of five fields were examined per slide then the mean was calculated for each rat.

### Statistical Analysis

Statistical Package for the Social Sciences (SPSS) version 16 was used to analyze the data. Study variables were compared using one-way analysis of variance (ANOVA) followed by Bonferroni *post hoc* test to avoid repeated comparisons. The results were presented as mean ± standard deviation. Significance was considered at *p* < 0.05.

## Results

### Behavioral Changes

**FIGURE 1:**
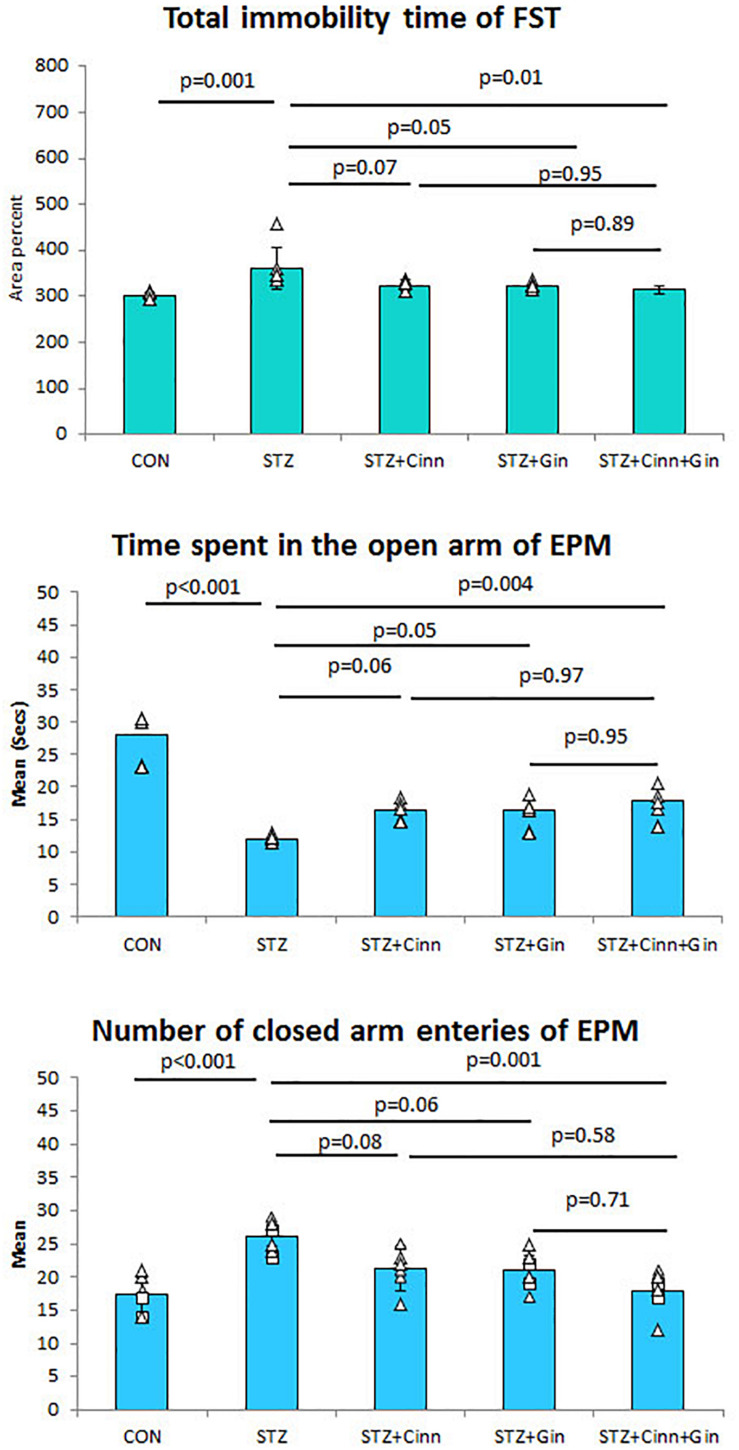
The immobility time of forced swimming test (FST), the mean time spent in the open arm of the elevated plus maze (EPM), and number of closed arm entries. CON, normal control; STZ, streptozotocin; Cinn, cinnamon; Gin, ginger. Data are presented as means ± SD, *n* = 6. Comparison between groups was done using one-way ANOVA test followed by the Bonferroni *post hoc* test.

To assess the antidepressant-like effect of the studied plants on the depressive like behavior of DM, both FST and EPM test were used. STZ-induced DM was associated with a significant increase (360.82 ± 46.89 vs. 300.59 ± 8.24, *p* < 0.001) in the immobility time compared to the control. Although the administration of ginger or cinnamon insignificantly reduced the immobility time, the combined administration of ginger and cinnamon could significantly (314.38 ± 7.97 vs. 360.82 ± 46.89, *p* = 0.01) reduce it compared to the untreated diabetic rats with insignificant difference between the three treated groups (Figure 1).

**FIGURE 2:**
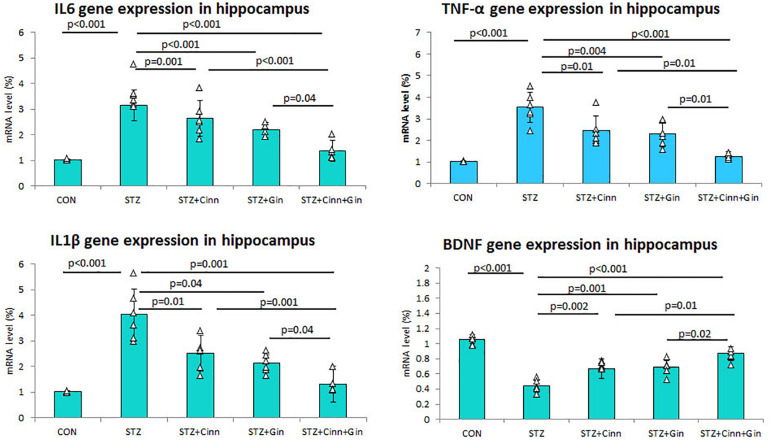
Relative expression of TNF-α, IL1β, and IL6 mRNA in hippocampus of the studied groups. CON, normal control; STZ, streptozotocin; Cinn, cinnamon; Gin, ginger. Data are presented as means ± SD, *n* = 6. Comparison between groups was done using one-way ANOVA test followed by the Bonferroni *post hoc* test.

Regarding the EPM test, it was noticed that the diabetic rats spent a significantly shorter time (11.99 ± 0.65 vs. 27.99 ± 3.9, *p* < 0.001) in the open arm compared to the control rats. Ginger and cinnamon, when separately administrated, insignificantly increased this time, while the combined administration of ginger and cinnamon significantly increased (17.80 ± 2.31 vs. 11.99 ± 0.65, *p* = 0.004) it compared to the untreated diabetic rats with insignificant difference between the three treated groups (Figure 2). The mean number of entries to the closed arm of the EPM was significantly higher (26.00 ± 2.37 vs. 17.33 ± 2.94, *p* < 0.001) in the untreated diabetic rats compared to the control. It was insignificantly lower in both ginger and cinnamon and significantly lower (17.83 ± 3.19 vs. 26.00 ± 2.37, *p* = 0.001) in the group that received combined administration, with insignificant difference between the three treated groups (Figure 1).

### Serum Corticosterone Level

Assessment of serum corticosterone level was conducted to confirm the depressive like behavior associated with DM. Diabetic rats in this study showed a significantly higher (11.59 ± 1.52 vs. 5.29 ± 0.71, *p* < 0.001) serum corticosterone level, while administration of ginger, cinnamon, or the combination of these significantly reduced it compared to the untreated diabetic rats. The combined administration of ginger and cinnamon resulted in a significant reduction in corticosterone level compared to the administration of ginger (8.77 ± 1.45 vs. 11.59 ± 1.52, *p* = 0.002) or cinnamon alone (9.12 ± 0.99 vs. 11.59 ± 1.52, *p* = 0.01) (Table 1).

**TABLE 1:** Effect of combined administration of *Zingiber officinale* and *Cinnamomum cassia* on the biochemical and behavioral parameters of the study.

Study variables	CON	STZ	STZ + Cinn	STZ + Gin	STZ + Cinn + Gin
Serum corticosterone (ng/ml)	5.29 ± 0.71	11.59 ± 1.52 *p* < 0.001	9.12 ± 0.99 *p*1 = 0.01	8.77 ± 1.45 *p*1 = 0.003 *p*2 = 0.98	6.19 ± 1.01 *p*1 < 0.001 *p*2 = 0.002 *p*3 = 0.01
Fasting blood glucose level in the serum (mg/dl) at the start of the experiment	79.57 ± 7.81	80.31 ± 3.91 *p* = 0.97	76.65 ± 8.79 *p*1 = 0.98	77.50 ± 6.94 *p*1 = 0.99 *p*1 = 0.97	74.50 ± 5.16 *p*1 = 0.96 *p*2 = 0.97 *p*3 = 0.98
Fasting blood glucose level in the serum (mg/dl) at the end of the experiment	80.91 ± 5.40	401.44 ± 49.05 *p* < 0.001	266.51 ± 29.32 *p*1 < 0.001	279.07 ± 52.45 *p*1 < 0.001 *p*2 = 0.98	192.34 ± 21.83 *p*1 < 0.001 *p*2 = 0.02 *p*3 = 0.003
Insulin level in serum (μIU/ml)	5.23 ± 0.41	2.11 ± 0.43 *p* < 0.001	3.72 ± 0.71 *p*1 = 0.002	3.82 ± 0.75 *p*1 < 0.001 *p*2 = 0.97	5.04 ± 0.87 *p*1 < 0.001 *p*2 = 0.02 p3 = 0.04
HOMA-IR	1.04 ± 0.06	2.08 ± 0.42 *p* = 0.02	2.46 ± 0.56 *p*1 = 0.98	2.86 ± 0.73 *p*1 = 0.12 *p*2 = 0.97	2.35 ± 0.49 *p*1 = 0.97 *p*2 = 0.99 *p*3 = 0.89
HOMA-β-cells	1.30 ± 0.17	0.11 ± 0.03 *p* < 0.001	0.28 ± 0.05 *p*1 = 0.08	0.31 ± 0.07 *p*1 = 0.03 *p*2 = 0.98	0.53 ± 0.13 *p*1 < 0.001 *p*2 = 0.004 *p*3 = 0.01
Total anti-oxidant capacity (TAC) in the serum (mM/dl)	296.50 ± 8.82	193.98 ± 9.59 *p* < 0.001	230.84 ± 11.97 *p*1 = 0.001	240.22 ± 15.12 *p*1 < 0.001 *p*2 = 0.97	280.12 ± 21.61 *p*1 < 0.001 *p*2 < 0.001 *p*3 = 0.001

*One-way analysis of variance was used to compare the studied groups followed by Bonferroni post hoc test. Results were presented in the form of mean ± standard deviation (SD). Significance was considered at p < 0.05.*

*p, significance vs. the control.*

*p1, significance vs. the STZ.*

*p2, significance vs. the STZ + Cinn.*

*p3, significance vs. the STZ + Gin.*

### Blood Glucose and Insulin Levels

The BGL was measured at the start and at the end of the experiment in order to assess the blood glucose lowering effect of the studied plants. It was noticed that the BGL was normal in all groups at the start of the experiment with insignificant changes between the studied groups. Administration of STZ resulted in a significant increase (401.44 ± 49.05 vs. 80.91 ± 5.40, *p* < 0.001) in BGL compared to the control, while the administration of ginger (77.50 ± 6.94 vs. 80.91 ± 5.40, *p* < 0.001), cinnamon (76.65 ± 8.79 vs. 80.91 ± 5.40, *p* < 0.001), or the combination of these (74.50 ± 5.16 vs. 80.91 ± 5.40, *p* < 0.001) induced a significant reduction compared to the untreated diabetic rats. The BGL was significantly lower in the rats that received the combined ginger and cinnamon compared to ginger-treated (*p* = 0.02) and cinnamon-treated rats (*p* = 0.003) (Table 1).

Regarding the serum insulin level, it was significantly reduced (2.11 ± 0.43 vs. 5.23 ± 0.41, *p* < 0.001) in STZ-treated rats compared to the control, while it was significantly increased in all the treated groups. The combined administration of combined ginger and cinnamon could significantly increase the insulin level (5.04 ± 0.87) compared to the administration of ginger (3.82 ± 0.75, *p* = 0.04) or cinnamon (vs. 3.72 ± 0.71, *p* = 0.02) alone.

### Blood Glucose Homeostasis

HOMA-IR was calculated to explore insulin resistance. It significantly increased (2.08 ± 0.42 vs. 1.04 ± 0.06, *p* = 0.02) in STZ-induced diabetic rats compared to the control, while it showed insignificant change in either ginger, cinnamon nor ginger plus cinnamon-treated rats compared to the STZ-induced diabetic rats with insignificant difference between the three treated groups (Table 1).

HOMA β-cell was calculated to assess β-cells function. It significantly decreased (0.11 ± 0.03 vs. 1.30 ± 0.17, *p* < 0.001) in STZ-induced diabetic rats compared to the control. Although, HOMA β-cell showed insignificant increase in cinnamon-treated rats, a significant increase was observed in ginger- (0.31 ± 0.07 vs. 0.11 ± 0.03, *p* = 0.03) and combined ginger and cinnamon-treated (0.53 ± 0.13 vs. 0.11 ± 0.03, *p* < 0.001) rats compared to the STZ-induced diabetic rats. HOMA β-cell significantly increased in the rats that received combined ginger and cinnamon compared to ginger-treated (*p* = 0.01) and cinnamon-treated rats (*p* = 0.004) (Table 1).

### Anti-oxidant Capacity

It was observed that STZ-induced diabetes in rats was associated with a significant decrease (193.98 ± 9.59 vs. 296.50 ± 8.82, *p* < 0.001) in rats treated in TAC in the serum compared to the control. Treatment with ginger (240.22 ± 15.12 vs. 193.98 ± 9.59, *p* < 0.001), cinnamon (230.84 ± 11.97 vs. 193.98 ± 9.59, *p* = 0.001), or the combination of these (280.12 ± 21.61 vs. 193.98 ± 9.59, *p* < 0.001) significantly increased TAC compared to STZ-induced diabetic rats. The TAC was significantly higher in the rats that received ginger plus cinnamon compared to ginger-treated (*p* = 0.001) and cinnamon-treated rats (*p* < 0.001) (Table 1).

### Gene Expression of Pro-inflammatory Cytokines

In order to assess the effect of the studied plants on the DM-associated inflammatory process, the hippocampal gene expression of pro-inflammatory cytokines was assessed using qRT-PCR. The mean expression of mRNA of IL-6 (3.16 ± 0.59 vs. 1.03 ± 0.03, *p* < 0.001), TNF-α (3.53 ± 0.71 vs. 1.03 ± 0.02, *p* < 0.001), and IL1β (4.03 ± 1.01 vs. 1.04 ± 0.02, *p* < 0.001) in the hippocampus was significantly upregulated in STZ-induced diabetic rats while it was significantly down-regulated in diabetic rats treated with ginger, cinnamon, or the combination of these. The level of mRNA of IL-6 (1.37 ± 0.35), TNF-α, and IL1β was significantly lower in the rats that received ginger plus cinnamon compared to ginger-treated (*p* = 0.04, *p* = 0.01, *p* = 0.04) and cinnamon-treated rats (*p* < 0.001, *p* = 0.01, *p* = 0.001) (Figure 2).

Regarding gene expression of BDNF in the hippocampus, it was noticed that mRNA of BDNF was significantly downregulated (0.44 ± 0.08 vs. 1.06 ± 0.06, *p* < 0.001) in STZ-induced diabetic rats while it was significantly upregulated in diabetic rats treated with ginger (0.69 ± 0.09 vs. 0.44 ± 0.08, *p* < 0.001), cinnamon (0.67 ± 0.13 vs. 0.44 ± 0.08, *p* = 0.002), or the combination of these (0.87 ± 0.09 vs. 0.44 ± 0.08, *p* < 0.001). mRNA of BDNF was significantly higher in the rats treated with ginger plus cinnamon compared to ginger-treated (*p* = 0.02) and cinnamon-treated rats (*p* = 0.01) (Figure 2).

### Histopathological Changes in the Hippocampus

**FIGURE 3:**
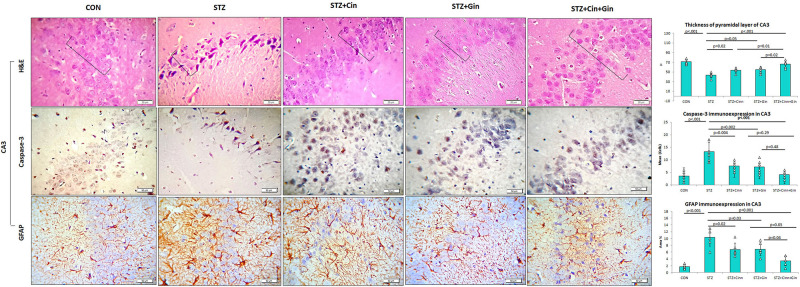
Photomicrographs of the CA3 region of the hippocampus in the studied groups. The practice indicates the pyramidal cell layer (H&E × 400). CON, normal control; STZ, streptozotocin; Cinn, cinnamon; Gin, ginger. Data are presented as means ± SD, *n* = 6. Comparison between groups was done using one-way ANOVA test followed by the Bonferroni *post hoc* test.

The histopathological structure of CA3 regions of the hippocampus and dentate gyrus (DG) and was assessed in this study as they are involved in controlling the mood. The CA3 region consists of three cell layers: the molecular, polymorphic, and pyramidal layers. The latter is formed of several layers of pyramidal nerve cells with basophilic cytoplasm and large open-face nuclei. The hippocampus of STZ-induced diabetic rats showed significantly thinner (42.17 ± 5.51, vs. 82.17 ± 5.53, *p* < 0.001) pyramidal cell layer compared to the control rats and many degenerated pyramidal cells with dark cytoplasm and nuclei. Treatment of diabetic rats with ginger, cinnamon, or the combination of these alleviated these pathological changes, reduced the number of degenerated cells, and significantly increased (*p* = 0.004, *p* = 0.002, *p* < 0.001), the thickness (61.67 ± 10.78, 60.00 ± 9.78, 76.33 ± 3.92 vs. 42.17 ± 5.51) of the pyramidal cell layer, respectively. The combination of ginger and cinnamon had the most significant effect compared to the other two treated groups (Figure 3).

**FIGURE 4:**
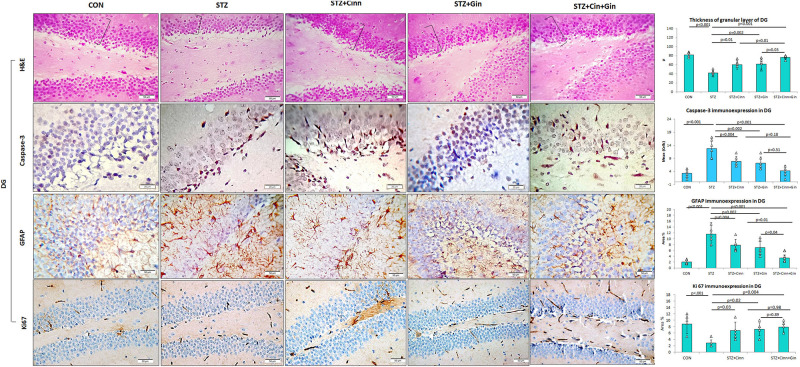
Photomicrographs of the dentate gyrus of the hippocampus in the studied groups. The practice indicates the granular cell layer (H&E × 400). DG, dentate gyrus; CON, normal control; STZ, streptozotocin; Cinn, cinnamon; Gin, ginger. Data are presented as means ± SD, *n* = 6. Comparison between groups was done using one-way ANOVA test followed by the Bonferroni *post hoc* test.

The DG of the hippocampus is formed mainly of polygonal cells with basophilic cytoplasm and vesicular nuclei named “the granular cells.” The DG of STZ-induced diabetic rats showed some degenerated granular cells with dark nuclei, which were less frequently observed in the treated rats. The thickness of the granular cell layer was significantly reduced (42.17 ± 5.51, vs. 82.17 ± 5.53, *p* < 0.001) in the hippocampus of diabetic rats compared to the control, while its thickness showed a significant increase in ginger (61.67 ± 10.78 vs. 42.17 ± 5.51, *p* = 0.002), cinnamon (60.00 ± 9.78 vs. 42.17 ± 5.51, *p* = 0.004), and combined ginger and cinnamon-treated groups (76.33 ± 3.9 vs. 42.17 ± 5.51, *p* < 0.001); the latter group showed the most significant effect (Figure 4). The apoptotic changes in the hippocampus were assessed using immunohistochemical staining with anti-capsase-3 antibodies. The number of caspase-3-positive cells was significantly increased (*p* < 0.001) in CA3 and DG of STZ-induced diabetic rats compared to the control, whereas it was significantly reduced in the three treated groups with insignificant difference between them (Figures 3, 4).

The integrity of astrocytes was immunohistochemically assessed using GFAP. It was noticed that GFAP immunoexpression was significantly increased in both CA3 (10.33 ± 2.6 vs. 1.7 ± 0.6, *p* < 0.001) and DG (11.5 ± 4.29 vs. 2.08 ± 0.7, *p* < 0.001) of STZ-induced diabetic rats when compared to the control. On the other hand, GFAP immunoexpression was significantly reduced in CA3 (6.85 ± 1.9, 6.77 ± 1.9, 3.47 ± 1.4 vs. 10.33 ± 2.6, *p* = 0.03, *p* = 0.02, *p* < 0.001) and DG (6.92 ± 2.5, 7.77 ± 2.00, 3.43 ± 1.43 vs. 11.5 ± 4.29, *p* = 0.004, *p* = 0.03, *p* < 0.001) of ginger, cinnamon or the combined ginger and cinnamon-treated groups, respectively, compared to the untreated diabetic group. GFAP immunoexpression was significantly reduced in CA3 and DG of combined ginger and cinnamon-treated groups compared to ginger- or cinnamon-treated group, respectively (Figures 3, 4).

Regarding ki67 immunoexpression in the hippocampus, it was noticed that it showed a significant reduction (2.83 ± 1.17 vs. 8.83 ± 2.79, *p* < 0.001) in DG of STZ-induced diabetic rats compared to the control, whereas it significantly increased in ginger, cinnamon, or the combined ginger and cinnamon-treated groups (7.17 ± 2.04, 6.83 ± 2.5, 7.83 ± 1.6 vs. 2.83 ± 1.17, *p* = 0.02, *p* = 0.03, *p* = 0.004), respectively, with insignificant difference between them (Figure 4).

## Discussion

Epidemiological and clinical studies have confirmed that the risk of some psychological disorders like depression, anxiety, cognitive impairments is higher among patients with diabetes compared to those without diabetes (Tu et al., 2017). Because of that, prevention and treatment of diabetes-induced neurological and behavioral disorders as well as improvement in the mental health of patients with diabetes, represents a major focus of research nowadays (Foley et al., 2018). In this context, this study was conducted to assess the possible synergistic anti-inflammatory, anti-hyperglycemic, and neuroprotective effect of *Cinnamomum cassia* and Z*ingiber officinale* and to explore the possible mechanisms behind this effect.

In the present study, it was noticed that administration of STZ resulted in a significant increase in the BGL and HOMA-IR associated with a significant decrease in serum insulin level and HOMA-β cells, compared to the control group, which indicated insulin resistance and reduced β-cell function, respectively. These results are in agreement with (Al-Qulaly et al., 2021) who have attributed this to β-cell destruction that occurs within 24 h after STZ administration and was associated with an inflammatory process with subsequent lymphocyte and macrophage infiltration. Administration of ginger, cinnamon, or the combination of these in the present study induced a significant reduction in BGL with the highest significance in the group that received the combination compared to ginger-treated and cinnamon-treated groups. Although the blood glucose level was significantly reduced in all treated groups compared to the untreated diabetic group, it was still significantly higher than that of the control group. Add to that, the serum insulin was still significantly lower than the control in both cinnamon- and ginger-treated groups. This might explain why the HOMA-IR and HOMA-B-cell were significantly different from the control.

The hypoglycemic effect of ginger was previously reported in many studies and it was associated with an increase in the activity of glycolytic enzymes: glucokinase, phosphofructokinase, and pyruvate kinase (Khandouzi et al., 2015). Ginger promotes glucose clearance in insulin-sensitive peripheral tissues, increases insulin release, and prevents STZ-induced oxidative stress through preventing lipid peroxidation (Alshathly, 2019). Regarding the effect of cinnamon on the BGL, it was reported that it reduced glucose transport and storage as a glycogen in the liver. It stimulates glucose transporters (GLUT4) for the entrance of glucose into the hepatic cells (glycogenesis) and adipocytes, leading to an increase in glycogen storage available for energy production (Mhammad et al., 2015). In this study, it was noticed that ginger, cinnamon, nor the combination of these could decrease insulin resistance as evident by HOMA-IR. On the other hand, administration of ginger alone or with cinnamon could significantly increase β-cell function as evidenced by increased serum insulin and HOMA-β. This is in line with some previous studies (Al-Qulaly et al., 2021; Ayuob et al., 2021). They added that ginger and cinnamon, administrated together, increase insulin secretion and increase tissue sensitivity to insulin.

STZ-induced DM was associated with a depressant-like behavior evident in the observed significant increase in the immobility time recorded in FST and confirmed by the EPM test. Although the administration of ginger or cinnamon insignificantly improved the assessed parameters of the behavioral tests, the combined administration of ginger and cinnamon could significantly improve them compared to the untreated diabetic rats with insignificant difference between the three treated groups. This finding is supported by a previous study which revealed that Eugenol, one of the compounds that exist in cinnamon, improves the symptoms of the neurodegenerative diseases such as Alzheimer, Parkinson, and depression and increases the expression of neurotropic factors in the hippocampus that lead to brain cell regeneration (Aryanezhad et al., 2021). Regarding ginger, it contains a different phenolic component, and it exhibited many bioactivities such as antioxidant and anti-anxiety (Nemati et al., 2021).

Dysfunction of the hypothalamic–pituitary–adrenocortical system (HPAS) and its regulation by the mechanism of glucocorticoid negative feedback cause the development of maladaptive states that can lead to severe functional disorders (depression and even death) (Vetrovoi et al., 2021). Diabetic rats in this study showed a significantly higher serum corticosterone level, known as stress marker, which was due to the hyperactivity of HPAS which mediates the stress response. Administration of ginger, cinnamon or the combination of these significantly reduced it compared to the untreated diabetic rats. This is in a partial agreement with (Yasmin, 2020) who reported that the administration of ginger honey and cocktail honey can reduce cortisol hormone levels and can be used as a supplement to deal with stress. In another study, cinnamon oil was reported to ameliorate deltamethrin-induced neurotoxicity, significantly improve behavioral performance, and reduce corticosterone level (Ahmed et al., 2021).

Regarding BDNF, it was reported to play a regulatory role in neural differentiation, synaptic plasticity, and cell death through two cell surface receptors (Aryanezhad et al., 2021). It was assessed in this study to explore its relation to diabetes-induced behavioral changes. We noticed a significant down-regulation in hippocampal mRNA of BDNF in STZ-induced diabetic rats, while it was significantly upregulated in diabetic rats treated with ginger, cinnamon, or the combination of these. This is supported by a recent study conducted by Nadimi et al. (2020) and reported that diabetes decreases BDNF levels by increasing oxidative stress or by other independent mechanisms of oxidative stress. Therefore, restoration of the redox state in diabetes may restore low BDNF levels, which can prevent complications associated with diabetes and the development of neurodegenerative diseases (Nadimi et al., 2020). Our findings regarding ginger- and cinnamon-induced up-regulation of BDNF expression are supported by a recent study conducted on the pre-frontal cortex of depressed rats (Aryanezhad et al., 2021).

The histopathological examination of both CA3 and DG of the hippocampus of STZ-induced diabetic rats revealed a significantly thinner pyramidal cell layer as well as many degenerated pyramidal cells, which pointed to atrophy in these regions. Similar regional changes were recorded by some researchers in previous studies (Amin et al., 2013; Faheem and El Askary, 2017). Among the mechanisms that explained diabetes-induced neurodegenerative changes is DM-associated hyperglycemia, which is a chronic endogenous stressor accompanied with increased hippocampal oxidative stress, by accelerating free radical generation that contributes to increased neuronal degeneration by inducing protein oxidation, DNA alterations, and lipids oxidation in cell membranes (Ceriello, 2003). Moreover, hyperglycemia elevates ROS in hippocampal neuronal cells resulting in a decrease in ATP activity and increase in Caspase-3 and 9 leading to Caspase-dependent apoptosis (Russell et al., 1999), which was evident in this study using immunohistochemical staining with anti-capsase-3 antibody, a marker of apoptosis. Treatment of diabetic rats with ginger, cinnamon, or the combination of these alleviated the pathological changes and significantly reduced apoptosis, and this was in accordance to the finding of El-Akabawy and El-Kholy (2014). They attributed this effect of ginger to its ability to down-regulate diabetes-induced oxidative distress, apoptosis, inflammation, gliosis, and achetylcholinesterase expression in different regions of the diabetic brain, in addition to its ability to improve diabetic brain-suppressed neurogenesis (El-Akabawy and El-Kholy, 2014). Another proposed mechanism that might contribute to neuronal loss in the diabetic rats of this study is reduced insulin which has been described to have a neuroprotective anti-apoptotic effect (Li et al., 2002).

STZ-induced DM was associated, in this study, with a significant increase in GFAP immunoexpression, an astrocyte specific marker, in both CA3 and DG, and this was supported by the findings of some previous studies (Faheem and El Askary, 2017). It was reported that any degenerative brain insult induced astrocyte proliferation and hypertrophy with increased GFAP production and resulted in severe astrogliosis, which may be considered as a compensatory process that play an important role in tissue repair after injury (Hashem et al., 2012). In this study, GFAP immunoreactivity was significantly reduced in both CA3 and DG of ginger, cinnamon, or the combined ginger and cinnamon-treated groups. Similar findings were recorded in cinnamon-fed diabetic rats (Puppala et al., 2019) as well as in ginger-fed diabetic rats (El-Akabawy and El-Kholy, 2014).

Ki-67 is an excellent marker for determining cellular proliferation (Irfannuddin et al., 2021). Hence, neuronal proliferation in the hippocampal DG was assessed using ki-67 immunoexpression. The latter was noticed to be significantly reduced in DG of STZ-induced diabetic rats, which is in agreement with those of the previous studies conducted on diabetic rats (El-Akabawy and El-Kholy, 2014). Although the mechanism of reduced neuronal proliferation is not well-known till now, evidence suggests that lack of insulin and/or hyperglycemia in the brain may be contributing factors (Zhang et al., 2008). Immunoexpression ki-67 was significantly upregulated in diabetic rats treated with ginger, cinnamon, and the combination of these. In a partial agreement with that, El-Akabawy and El-kholy have reported that ginger antagonized the diabetes-induced suppression of neurogenesis induced by DM-associated oxidative stress (El-Akabawy and El-Kholy, 2014).

In order to explore the link between DM-associated neural changes and the inflammatory process, the hippocampal gene expression of pro-inflammatory cytokines was assessed using qRT-PCR. The expression of mRNA of IL-6, TNF-α, and IL1β in the hippocampus was significantly upregulated in STZ-induced diabetic rats, while in this study, it was significantly down-regulated in diabetic rats treated with ginger, cinnamon, or the combination of these with the maximum effect observed in the latter group. It was reported that the free oxygen radicals and advanced glycation products (AGEs) prompted by hyperglycemia can enhance the production of inflammatory cytokines including TNF-α (El-Akabawy and El-Kholy, 2014; Edrees et al., 2017). Moreover, TNF−α plays a major role in the activation of caspases which are important in the induction of cell apoptosis (Li et al., 2016). Increased Caspase-3 immunoexpression was also evident in this study. Reduced TNF−α after the administration of ginger or cinnamon was supported by the findings of previous studies (Al-Qulaly et al., 2021).

In the present study, it was observed that STZ-induced diabetes in rats was associated with a significant decrease in serum TAC. It was reported that increased oxidative stress, which contributes substantially to the pathogenesis of diabetic complications, is the consequence of either enhanced ROS production or attenuated ROS scavenging capacity (Shanmugam et al., 2011). Our results were in line with those of the earlier studies which revealed that the cerebral cortex, cerebellum, hippocampus, and hypothalamus of diabetic rats have lower anti-oxidative defense system activity compared to non-diabetic rats (El-Missiry et al., 2004). In this study, ginger and cinnamon, administrated separately or together, significantly increased the TAC in diabetic rats. In previous studies, ginger-induced improvement in diabetes patients and experimental animals was explained by the powerful scavenging effect of free radicals induced by active constituents of ginger like gingerol, vallinoids, paradol, and zingerone (El-Ghawet et al., 2019; Nemati et al., 2021). Cinnamon also has potent antioxidant activities because it is rich in procyanidins and catechins, which reduce oxidative stress and prevent β-cell damage, and this may be explain the anti-diabetic activity of cinnamon (Al-Waili et al., 2017). Administration of ginger and cinnamon together resulted in a synergism in their antioxidant effect, which might explain the significant anti-hyperglycemic and anti-inflammatory effect compared to the single administration of either of them.

In conclusion, in this study, the synergism between ginger and cinnamon regarding the anti-hyperglycemic, insulin-enhancing, antioxidant, anti-inflammatory, antidepressant-like, and neuroprotective effect was studied and confirmed. The antidepressant-like effect of ginger and cinnamon might be directly mediated through the up-regulation of BDNF and down-regulation of inflammatory cytokines and indirectly mediated through their anti-hyperglycemic and antioxidant activities. Further research for exploring the detailed mechanism of the synergistic antidepressant-like effect of ginger and cinnamon is encouraged, and testing this effect in diabetic patients is recommended.

## Data Availability Statement

The original contributions presented in the study are included in the article/Supplementary Material, further inquiries can be directed to the corresponding author/s.

## Ethics Statement

The animal study was reviewed and approved by the Biomedical Research Ethics Committee,” Faculty of Medicine, King Abdulaziz University.

## Author Contributions

HA, AB, and ZM supervised the experiment, collected data for the investigation, and participated in result interpretation. MB, HB, OR, SA, and NA designed the experimental protocol, were involved in the implementation of the overall study, performed the statistical analysis of the study, researched the data, and wrote the manuscript. All authors contributed to the revision of the manuscript and approval of the final manuscript.

## Conflict of Interest

The authors declare that the research was conducted in the absence of any commercial or financial relationships that could be construed as a potential conflict of interest. The reviewer AO declared a shared affiliation, with no collaboration, with one of the authors, NA, to the handling editor at the time of the review.

## Publisher’s Note

All claims expressed in this article are solely those of the authors and do not necessarily represent those of their affiliated organizations, or those of the publisher, the editors and the reviewers. Any product that may be evaluated in this article, or claim that may be made by its manufacturer, is not guaranteed or endorsed by the publisher.
